# Factors associated to prevalence and treatment of carbapenem-resistant Enterobacteriaceae infections: a seven years retrospective study in three tertiary care hospitals

**DOI:** 10.1186/s12941-018-0267-8

**Published:** 2018-03-23

**Authors:** Feng Pang, Xiu-Qin Jia, Qi-Gang Zhao, Yi Zhang

**Affiliations:** 1 Department of Clinical Laboratory, Qilu Hospital of Shandong University, Jinan, 250012 Shandong People’s Republic of China; 2 Department of Clinical Laboratory, Liaocheng People’s Hospital, Liaocheng, 252000 Shandong People’s Republic of China; 3 Department of Clinical Pharmacy, Liaocheng People’s Hospital, Liaocheng, 252000 Shandong People’s Republic of China

**Keywords:** Antibiotics, Carbapenem-resistant Enterobacteriaceae, Infection, Outcome, Treatment

## Abstract

**Background:**

The increasing incidence of carbapenem-resistant *Enterobacteriaceae* (CRE), has resulted in a difficult problem in the current clinical anti-infective treatment. We performed a retrospective analysis of prevalence and treatment for CRE infections patients.

**Methods:**

This study was conducted in three tertiary care hospitals from January 1, 2010 to December 30, 2016. Baseline data, treatment, and outcomes were collected in patients with ventilator-associated bacterial pneumonia (VABP), bacteremia, complicated urinary tract infection (cUTI)/acute pyelonephritis (AP), hospital-acquired bacterial pneumonia (HABP), superficial wound infection (SWI), biliary tract infection (BTI), deep wound infection (DWI) and sterile body fluids infection (SBFI) due to CRE.

**Results:**

One hundred twenty-four cases of CRE infection were identified: 31 VABP, 22 bacteremia, 18 cUTI/AP, 16 HABP, 16 SWI, 9 BTI, 7 DWI and 5 SBFI. The patient population had significant immunocompromised (33 of 124, 26.6%) and severe sepsis (43 of 124, 34.7%). The most common CRE pathogens were *Klebsiella pneumoniae* (84 of 124, 67.7%) and *Enterobacter cloacae* (24 of 124, 19.4%). And the production of IMP-type carbapenemase was the main antibiotic resistance mechanism. The majority of patients to take monotherapy for empiric therapy and dual therapy for direct treatment. Outcomes were universally poor (28-day mortality was 22.6%, 28 of 124) across all sites of infection.

**Conclusions:**

We identified a large number of cases of CRE infection in 7 years from different parts, most of these pathogens have been confirmed to produce IMP-type carbapenemases. The retrospective analysis of cases of such bacterial infections will help to control future infections of these pathogens. Despite the high mortality rate, we still found that the selection of quinolone antibiotics can be effective in the treatment of CRE producing IMP type enzymes.

## Background

Since 2000, spread of community-acquired enterobacterial isolates that produce extended-spectrum β-lactamases (ESBLs) has been reported worldwide [1]. Carbapenems have become the best and last drug for treating this strain of infection. However, the frequent use of carbapenems has contributed to the increase of carbapenem resistance by Enterobacteriaceae [2, 3]. In countries such as the United States, Germany and Greece, carbapenem-resistant Enterobacteriaceae (CRE) had emerged and the dissemination of these multidrug-resistant pathogens had become a problem in clinical care of patients and in public health [4–6]. In China, two cases of pulmonary infection due to carbapenem-resistant *Klebsiella pneumoniae* strains producing *bla*_*IMP*-*4*_ and *armA* were found in Hangzhou [7]. A subsequent multisectoral study confirmed the presence of CRE strains in several districts and hospitals in China [8]. Due to their resistance to most available antimicrobial agents, invasive infections with these organisms have been associated with high rates of morbidity and mortality [9, 10], Studying the characteristics of clinical infection with CRE is conducive to the targeted treatment of this kind of infection. Our previous study has confirmed the presence of a large amount of IMP-producing carbapenemase CRE in three hospitals of Liaocheng city, in China. This study mainly focuses on the clinical data of pre-CRE strains in order to obtain the measures to control and treat infection of such strains.

## Methods

### Setting and study design

This was a retrospective analysis of patients with CRE infection at three large comprehensive tertiary hospital in Liaocheng city (Liaocheng People’s Hospital, Liaocheng Chinese Medicine hospital, Liaocheng Third People’s Hospital). The three hospitals can represent the whole city of bacteria infection. Patients were included if CRE pathogens identified from respiratory, blood, urine, superficial pus, bile, deep pus or sterile body fluid samples from January 1, 2010 to December 30, 2016. Any patient who had clinical evidence of ventilator-associated bacterial pneumonia (VABP), bacteremia, complicated urinary tract infection (cUTI)/acute pyelonephritis (AP), hospital-acquired bacterial pneumonia (HABP), superficial wound infection (SWI), biliary tract infection (BTI), deep wound infection (DWI) and sterile body fluids infection (SBFI) requiring antimicrobial treatment was eligible for inclusion. The same patient with recurrent infections were defined only once infection. In cases of concurrent infection, patients were classified according to the primary CRE infection. Patients’ data were compiled until death or hospital discharge.

Three hospital conducted susceptibility of Enterobacteriaceae according to Clinical and Laboratory Standards Institute (CLSI) breakpoint definitions. Carbapenem resistance was defined according to CLSI M100S, 26th [11]. The resistance mechanisms of CRE was proved by modified Hodge test and sequencing as our previous studies [12].

### Clinical definitions

A prior culture positive for CRE was defined as a culture for CRE obtained from any site within 90 days before the qualifying CRE infection. In view of some strains were isolated from the pediatric ward, the data for pediatric patients (age ≤ 1 year) have been excluded when calculating mean and standard deviation of the age. Surgical wards included hepatobiliary surgery, orthopedics, urology surgery, gastrointestinal surgery and anorectal surgery. Pediatric wards included general pediatric, pediatric intensive care unit and neonatal intensive care unit. Medical wards included respiratory, neurology, urology, hematology, cardiology and gastroenterology. Other wards included dermatology, otolaryngology, burns, radiotherapy and emergency department. Immunocompromised conditions included hematologic malignancy, prior bone marrow transplant, or received immunosuppressive therapy, such as cancer chemotherapy, antirejection medications for transplantation, or long-term (≥ 2 weeks) use of systemic steroids. Neutropenia was defined as < 500 neutrophils/mm^3^ for adults patients, and < 750 neutrophils/mm^3^ for pediatric patients. Severe sepsis was defined as infection associated with any of the following: hypotension (systolic blood pressure [SBP] ≤ 90 mmHg or a decrease in SBP of ≥ 40 mmHg from baseline unresponsive to fluid challenge), hypothermia (core temperature < 35.6 °C), or disseminated intravascular coagulation (DIC) as evidenced by prothrombin time or partial thromboplastin time 2× the upper limit of normal or platelets less than 50% of the lower limit of normal. Septic shock was defined as infection associated with hypotension (SBP ≤ 90 mmHg or a decrease in SBP of ≥ 40 mmHg). Clinical cure was defined as resolution of signs and symptoms, no further antibiotics for the treatment of CRE infection were needed [13].

Empiric treatment was defined as any antimicrobial treatment that began before the date on which all susceptibility data were available for CRE pathogen. Directed treatment was defined as any antimicrobial treatment that began on or after the date on which all susceptibility data were available for CRE pathogen. Empiric and directed therapies were categorized according to the number of agents with in vitro activity for at least 3 calendar days. Microbiological eradication was defined as absence of CRE on follow-up cultures.

## Results

### Infection types and patient characteristics

One hundred twenty-four cases of CRE infection were identified: 31 VABP, 22 bacteremia, 18 cUTI/AP, 16 HABP, 16 SWI, 9 BTI, 7 DWI and 5 SBFI, and surgical wards and intensive care unit (ICU) were the most frequent residence of pathogen. The average age of patients was 56 years (exclusion of pediatric patients age ≤ 1 year) with a male predominance (66.9%) across all disease types. VABP appeared a high incidence (64.5%) in the ICU, while the cUTI/AP, SWI, BTI, DWI and SBFI have a high proportion in the surgical ward, accounting for 61.1, 75.0, 88.9, 57.1 and 80.0% respectively. Thirty-four patients (27.4%) had a prior positive culture for CRE within the preceding 90 days, and average duration of hospitalization before CRE infection was 9.7 days. The patient population had significant immunocompromised (33 of 124, 26.6%) and severe sepsis (43 of 124, 34.7%). Patients with diabetes were more likely to isolate CRE from VABP, bacteremia and cUTI/AP. Infected patients with VABP and bacteremia associated with multiple comorbidities and serious presentations frequently. It is worth noting that among the 31 patients with VABP, 10 (32.3%) had concurrent CRE bacteremia (Table 1).

**Table 1:** Characteristics of the study cohort stratified by type of infection

Variable	Number (%) of patients								
	Infection type								
	VABP (N = 31)	Bacteremia (N = 22)	cUTI/AP (N = 18)	HABP (N = 16)	SWI (N = 16)	BTI (N = 9)	DWI (N = 7)	SBFI (N = 5)	All (N = 124)
Demographic variables									
Male sex	23 (74.2%)	14 (63.6%)	8 (44.4%)	12 (75.0%)	9 (56.3%)	6 (66.7%)	6 (85.7%)	5 (100%)	83 (66.9%)
Age, years, mean (SD)^a^	60.4 (20.2)	48.1 (26.0)	59.4 (15.5)	72.9 (14.5)	52.8 (17.3)	55.6 (8.3)	57.6 (11.8)	40.8 (8.4)	56.1 (19.3)
Department of residence^b^									
ICU	20 (64.5%)	6 (27.3%)	2 (11.1%)	0	0	1 (11.1%)	1 (14.3%)	1 (20.0%)	31 (25.0%)
Pediatric wards	11 (35.5%)	6 (27.3%)	0	8 (50.0%)	0	0	0	0	25 (20.2%)
Surgical wards	0	3 (13.6%)	11 (61.1%)	0	12 (75.0%)	8 (88.9%)	4 (57.1%)	4 (80.0%)	42 (33.9%)
Medical wards	0	7 (31.8%)	4 (22.2%)	6 (37.5%)	2 (12.5%)	0	0	0	19 (15.3%)
Other wards	0	0	1 (5.5%)	2 (12.5%)	2 (12.5%)	0	2 (28.6%)	0	7 (5.6%)
Specific risk factors									
Prior culture positive for CRE	12 (38.7%)	8 (36.4%)	2 (11.1%)	2 (12.5%)	1 (6.3%)	3 (33.3%)	0	1 (20.0%)	34 (27.4%)
Duration of hospitalization before CRE infection, mean (SD)	10.8 (6.3)	11.5 (4.7)	6.6 (3.4)	9.6 (5.9)	8.8 (5.0)	7.1 (3.3)	11.9 (5.3)	9.8 (1.9)	9.7 (4.6)
Immunocompromised condition^c^	10 (32.3%)	6 (27.3%)	4 (22.2%)	5 (31.3%)	4 (25.0%)	1 (11.1%)	2 (28.6%)	1 (20.0%)	33 (26.6%)
Presence of neutropeniad^d^	2 (6.5%)	3 (13.6%)	0	1 (6.3%)	0	0	0	0	6 (4.8%)
Prior transplantation	2 (6.5%)	2 (9.1%)	0	0	0	1 (11.1%)	0	0	5 (4.0%)
Comorbidities									
Diabetes mellitus	6 (19.4%)	5 (22.7%)	7 (38.9%)	2 (12.5%)	3 (18.8%)	0	1 (14.3%)	1 (20.0%)	25 (20.2%)
Solid tumor	3 (9.7%)	2 (9.1%)	3 (16.7%)	2 (12.5%)	4 (25.0%)	2 (22.2%)	1 (14.3%)	2 (40.0%)	19 (15.3%)
Chronic renal insufficiency	5 (16.1%)	4 (18.2%)	5 (27.8%)	0	3 (18.8%)	0	1 (14.3%)	0	18 (14.5%)
Heart failure	5 (16.1%)	4 (18.2%)	0	2 (12.5%)	0	0	0	1 (20.0%)	12 (9.7%)
Hematologic malignancy	1 (3.2%)	3 (13.6%)	0	0	0	0	0	0	4 (3.2%)
Concurrent bacteremia	10 (32.3%)	NA	2 (11.1%)	4 (25.0%)	1 (6.3%)	3 (33.3%)	3 (42.9%)	2 (40.0%)	NA
Presentation with severe sepsis^e^	20 (64.5%)	10 (45.5%)	2 (11.1%)	4 (25.0%)	1 (6.3%)	2 (22.2%)	2 (28.6%)	2 (40.0%)	43 (34.7%)
Presentation with septic shock^f^	16 (51.6%)	8 (36.4%)	1 (5.5%)	2 (12.5%)	1 (6.3%)	2 (22.2%)	2 (28.6%)	2 (40.0%)	34 (27.4%)

*AP* acute pyelonephritis, *BTI* biliary tract infection, *CRE* carbapenem-resistant Enterobacteriaceae, *cUTI* complicated urinary tract infection, *DIC* disseminated intravascular coagulation, *DWI* deep wound infection, *HABP* hospital-acquired bacterial pneumonia, *ICU* intensive care unit, *NA* not applicable, *SBFI* sterile body fluids infection, *SBP* systolic blood pressure, *SD* standard deviation, *SWI* superficial wound infection, *VABP* ventilator-associated bacterial pneumonia

^a^The data for pediatric patients (age ≤ 1 year) have been excluded when calculating mean and SD of the age

^b^Surgical wards include hepatobiliary surgery, orthopedics, urology surgery, gastrointestinal surgery and anorectal surgery. Pediatric wards include general pediatric, pediatric intensive care unit and neonatal intensive care unit. Medical wards include respiratory, neurology, urology, hematology, cardiology and gastroenterology. Other wards include dermatology, otolaryngology, burns, radiotherapy and emergency department

^c^Immunocompromised conditions included hematologic malignancy, prior bone marrow transplant, or received immunosuppressive therapy, such as cancer chemotherapy, antirejection medications for transplantation, or long-term (≥ 2 weeks) use of systemic steroids

^d^Neutropenia was defined as < 500 neutrophils/mm^3^ for adults patients, and < 750 neutrophils/mm^3^ for pediatric patients

^e^Severe sepsis was defined as infection associated with any of the following: hypotension (SBP ≤ 90 mmHg or a decrease in SBP of ≥ 40 mmHg), hypothermia (core temperature < 35.6 °C), or DIC as evidenced by prothrombin time or partial thromboplastin time 2× the upper limit of normal or platelets less than 50% of the lower limit of normal [3]

^f^Septic shock was defined as infection associated with hypotension (SBP ≤ 90 mmHg or a decrease in SBP of ≥ 40 mmHg) [3]

### Characteristics of infecting isolates

The most common CRE pathogens were *K. pneumoniae*, other isolates included *Enterobacter cloacae*, *Escherichia coli* and *Klebsiella oxytoc*a. However, *E. cloacae* was the commonly isolate in SWI (Table 2). Confirmed by sequencing, 54 strains of CRE were produced IMP-4 type carbapenemase, which included 42 *K. pneumoniae*, 8 *E. cloacae* and 4 *K. oxytoca*. While 40 strains of CRE were confirmed to produce IMP-8 carbapenemase, including 20 *K. pneumoniae*, 12 *E. cloacae*, 6 *E. coli* and 2 *K. oxytoca*. The other 30 strains did not detect the carbapenemase genetype (Table 3).

**Table 2:** Microbiology of carbapenem-resistant Enterobacteriaceae isolate by type of infection

Pathogen	VABP (n = 31) n (%)	Bacteremia (n = 22) n (%)	cUTI/AP (n = 18) n (%)	HABP (n = 16) n (%)	SWI (n = 16) n (%)	BTI (n = 9) n (%)	DWI (n = 7) n (%)	SBFI (n = 5) n (%)	All (n = 124) n (%)
*Klebsiella pneumoniae*	27 (87.1%)	18 (81.8%)	10 (55.6%)	11 (68.8%)	3 (18.8%)	4 (44.4%)	6 (85.7%)	5 (100%)	84 (67.7%)
*Enterobacter cloacae*	3 (9.7%)	2 (9.1%)	3 (16.7%)	2 (12.5%)	10 (62.5%)	4 (44.4%)	0	0	24 (19.4%)
*Escherichia coli*	0	2 (9.1%)	5 (27.8%)	2 (12.5%)	1 (6.3%)	0	0	0	10 (8.1%)
*Klebsiella oxytoca*	1 (3.2%)	0	0	1 (6.3%)	2 (12.5%)	1 (11.1%)	1 (14.3%)	0	6 (4.8%)

*AP* acute pyelonephritis, *BTI* biliary tract infection, *cUTI* complicated urinary tract infection, *DWI* deep wound infection, *HABP* hospital-acquired bacterial pneumonia, *SBFI* sterile body fluids infection, *SWI* superficial wound infection, *VABP* ventilator-associated bacterial pneumonia

**Table 3:** Resistance mechanisms of infecting isolate by pathogen type

Pathogen	IMP-4	IMP-8	Not detected	All
*Klebsiella pneumoniae*	42	20	22	84
*Enterobacter cloacae*	8	12	4	24
*Escherichia coli*	0	6	4	10
*Klebsiella oxytoca*	4	2	0	6
Total	54	40	30	124

All CRE isolates showed resistance to ceftriaxone, ceftazidime, cefepime and other cephalosporins antimicrobial agents. More than 80% of the isolates were nonsusceptible (intermediate or resistant) to tobramycin (93.5%) and trimethoprim-sulfamethoxazole (88.7%). The nonsusceptible rate of CRE to gentamicin was 56.5%, while 46.3% nonsusceptible to piperacillin/tazobactam, 38.7% nonsusceptible to ciprofloxacin, 20.2% nonsusceptible to tigecycline and 7.3% nonsusceptible to polymyxin B.

### Treatment and outcomes

Among the 124 patients included, a majority (110 of 124, 88.7%) received empiric therapy with antibiotic against gram-negative pathogens. Approximately half of the patients (63 of 124, 50.8%) were received empiric monotherapy, including VABP (13 of 31, 41.9%), bacteremia (10 of 22, 45.5%), cUTI/AP (12 of 18, 66.7%), HABP (8 of 16, 50.0%), SWI (11 of 16, 68.8%), DWI (4 of 7, 57.1%) and SBFI (3 of 5, 60.0%). BTI patients were most likely to receive empiric therapy with two agents with gram-negative activity (4 of 9, 44.4%). Despite this, only 35 antibiotic subjects (28.2%) had in vitro activity against the CRE isolates (Table 4). The most common classes of empiric antimicrobials were β-lactam/β-lactamase inhibitor combinations (e.g., ampicillin, ampicillin-sulbactam, amoxicillin-clavulanic acid), cephalosporins (e.g., ceftriaxone, cefotaxime) and carbapenems (e.g., imipenem, meropenem).

**Table 4:** Empiric antimicrobial agents stratified by type of infection

Empiric antimicrobial therapy	Number (%) of patients								
	Infection type								
	VABP (N = 31)	Bacteremia (N = 22)	cUTI/AP (N = 18)	HABP (N = 16)	SWI (N = 16)	BTI (N = 9)	DWI (N = 7)	SBFI (N = 5)	All (N = 124)
No treatment	0	1 (4.5%)	4 (22.2%)	1 (6.3%)	3 (18.8%)	2 (22.2%)	1 (14.3%)	0	12 (9.7%)
No Gram-negative coverage	0	0	1 (5.6%)	0	1 (6.3%)	0	0	0	2 (1.6%)
Gram-negative therapy	31 (100%)	21 (%)	13 (72.2%)	15 (93.8%)	12 (75%)	7 (77.8%)	6 (85.7%)	5 (100%)	110 (88.7%)
Monotherapy	13 (41.9%)	10 (45.5%)	12 (66.7%)	8 (50.0%)	11 (68.8%)	2 (22.2%)	4 (57.1%)	3 (60.0%)	63 (50.8%)
Dual therapy	9 (29.0%)	8 (36.4%)	1 (5.6%)	5 (31.3%)	1 (6.25%)	4 (44.4%)	2 (28.6%)	2 (40.0%)	32 (25.8%)
Three drug combinations	6 (19.4%)	2 (9.1%)	0	2 (12.5%)	0	1 (11.1%)	0	0	11 (8.9%)
Four and more drug combinations	3 (9.7%)	1 (4.5%)	0	0	0	0	0	0	4 (3.2%)
Number active agents									
No active agent	20 (64.5%)	15 (68.2%)	7 (38.9%)	13 (81.3%)	6 (37.5%)	5 (55.6%)	5 (71.4%)	4 (80.0%)	75 (60.5%)
One active agent	9 (29.0%)	5 (22.7%)	6 (33.3%)	2 (12.5%)	6 (37.5%)	2 (22.2%)	1 (14.3%)	1 (20.0%)	32 (25.8%)
Two active agents	2 (6.45%)	1 (4.5%)	0	0	0	0	0	0	3 (2.4%)

*AP* acute pyelonephritis, *BTI* biliary tract infection, *cUTI* complicated urinary tract infection, *DWI* deep wound infection, *HABP* hospital-acquired bacterial pneumonia, *SBFI* sterile body fluids infection, *SWI* superficial wound infection, *VABP* ventilator-associated bacterial pneumonia

The majority of patients (112 of 124, 90.3%) ultimately received directed antimicrobial gram-negative therapy. The proportion of monotherapy, dual therapy, three drug combinations and four and more drug combinations were 25.0, 40.3, 21.0 and 4.0% respectively. In vitro activity against CRE isolate of one agent, two agents, three agents and four agents were 57.3% (71 of 124), 21.8% (27 of 124), 1.6% (2 of 124) and 0.8% (1 of 124) (Table 5). The most common directed antimicrobials were quinolones, piperacillin/tazobactam, tigecycline, carbapenems, polymyxins and aminoglycosides. Nevertheless, we were unable to identify a best available monotherapy or combination regimen against CRE due to the large number of different regimens used in the treatment of CRE infections.

**Table 5:** Directed antimicrobial agents stratified by type of infection

Directed antimicrobial therapy	Number (%) of patients								
	Infection type								
	VABP (N = 31)	Bacteremia (N = 22)	cUTI/AP (N = 18)	HABP (N = 16)	SWI (N = 16)	BTI (N = 9)	DWI (N = 7)	SBFI (N = 5)	All (N = 124)
No treatment	2 (6.5%)	1 (4.5%)	1 (5.6%)	1 (6.3%)	3 (18.8%)	1 (11.1%)	1 (14.3%)	0	10 (8.1%)
No Gram-negative coverage	0	0	0	0	1 (6.3%)	0	1 (14.3%)	0	2 (1.6%)
Gram-negative therapy	29 (93.5%)	21 (95.5%)	17 (94.4%)	15 (93.8%)	12 (75.0%)	8 (88.9%)	5 (71.4%)	5 (100%)	112 (90.3%)
Monotherapy	3 (9.7%)	1 (4.5%)	12 (66.7%)	5 (31.3%)	6 (37.5%)	2 (22.2%)	1 (14.3%)	1 (20.0%)	31 (25.0%)
Dual therapy	13 (41.9%)	8 (36.4%)	5 (27.8%)	8 (50.0%)	6 (37.5%)	5 (55.6%)	3 (42.9%)	2 (40.0%)	50 (40.3%)
Three drug combinations	10 (32.3%)	10 (45.5%)	0	2 (12.5%)	0	1 (11.1%)	1 (14.3%)	2 (40.0%)	26 (21.0%)
Four and more drug combinations	3 (9.7%)	2 (9.1%)	0	0	0	0	0	0	5 (4.0%)
Number active agents									
No active agent	3 (9.7%)	3 (13.6%)	1 (5.6%)	2 (12.5%)	1 (6.3%)	0	1 (14.3%)	0	11 (8.9%)
One active agent	16 (51.6%)	12 (54.5%)	15 (83.3%)	10 (62.5%)	8 (50.0%)	4 (44.4%)	3 (42.9%)	3 (60.0%)	71 (57.3%)
Two active agents	9 (29.0%)	4 (18.2%)	1 (5.6%)	3 (18.8%)	3 (18.8%)	4 (44.4%)	1 (14.3%)	2 (40.0%)	27 (21.8%)
Three active agents	1 (3.2%)	1 (4.5%)	0	0	0	0	0	0	2 (1.6%)
Four active agents	0	1 (4.5%)	0	0	0	0	0	0	1 (0.8%)

*AP* acute pyelonephritis, *BTI* biliary tract infection, *cUTI* complicated urinary tract infection, *DWI* deep wound infection, *HABP* hospital-acquired bacterial pneumonia, *SBFI* sterile body fluids infection, *SWI* superficial wound infection, *VABP* ventilator-associated bacterial pneumonia

The average duration of hospitalization for CRE infection was 11.3 days, while the average duration of ICU stay was 5.1 days. Majority of patients (88 of 124, 71.0%) achieved clinical cure and 81 of 124 (65.3%) achieved microbiologic eradication at the completion of antibiotic treatment. Twenty-eight patients (22.6%) died within 28 days across all sites of the CRE infection. Clinical cure and 28-day mortality of six patients could not be traced due to transfer to other hospital. And nine patients did not carry out microbiological testing after the condition improved, thus CRE eradication of those patients could not retrospectively observe (Table 6).

**Table 6:** Outcomes of infections due to carbapenem-resistant Enterobacteriaceae by type of infection

Outcome	Number (%) of patients								
	Infection type								
	VABP (N = 31)	Bacteremia (N = 22)	cUTI/AP (N = 18)	HABP (N = 16)	SWI (N = 16)	BTI (N = 9)	DWI (N = 7)	SBFI (N = 5)	All (N = 124)
Duration of hospitalization for CRE infection^a^ (mean ± SD)	15.8 (14.3)	14.6 (7.9)	6.7 (9.4)	10.5 (4.5)	9.3 (3.2)	7.6 (5.4)	8.3 (4.9)	10.4 (2.1)	11.3 (9.7)
Duration of ICU stay^a^ (mean ± SD)	8.4 (6.9)	9.2 (8.4)	2.3 (3.9)	3.5 (2.9)	1.7 (4.4)	1.9 (2.4)	2.4 (2.4)	2.0 (2.8)	5.1 (4.2)
Clinical cure, n (%)									
Yes	18 (58.1%)	10 (45.5%)	16 (88.9%)	11 (68.8%)	15 (93.8%)	9 (100%)	5 (71.4%)	4 (80.0%)	88 (71.0%)
No	11 (35.5%)	11 (50.0%)	2 (11.1%)	4 (25.0%)	1 (6.3%)	0	1 (14.3%)	1 (20.0%)	30 (24.2%)
Unknown	2 (6.5%)	1 (4.5%)	0	1 (6.3%)	0	0	1 (14.3%)	0	6 (4.8%)
CRE eradicated, n (%)									
Yes	15 (48.4%)	12 (54.5%)	16 (88.9%)	10 (62.5%)	10 (62.5%)	9 (100%)	5 (71.4%)	4 (80.0%)	81 (65.3%)
No	12 (38.7%)	9 (40.9%)	1 (5.6%)	4 (25.0%)	6 (37.5%)	0	1 (14.3%)	1 (20.0%)	34 (27.4%)
Unknown	4 (12.9%)	1 (4.5%)	1 (5.6%)	2 (12.5%)	0	0	1 (14.3%)	0	9 (7.3%)
28-day mortality, n (%)									
Yes	10 (32.3%)	10 (45.5%)	1 (5.6%)	4 (25.0%)	1 (6.3%)	0	1 (14.3%)	1 (20.0%)	28 (22.6%)
No	19 (61.3%)	11 (50.0%)	17 (94.4%)	10 (62.5%)	15 (93.8%)	9 (100%)	5 (71.4%)	4 (80.0%)	90 (72.6%)
Unknown	2 (6.5%)	1 (4.5%)	0	2 (12.5%)	0	0	1 (14.3%)	0	6 (4.8%)

*AP* acute pyelonephritis, *BTI* biliary tract infection, *CRE* carbapenem-resistant Enterobacteriaceae, *cUTI* complicated urinary tract infection, *DWI* deep wound infection, *HABP* hospital-acquired bacterial pneumonia, *ICU* intensive care unit, *SBFI* sterile body fluids infection, *SD* standard deviation, *SWI* superficial wound infection, *VABP* ventilator-associated bacterial pneumonia

^a^Duration of hospitalization for CRE infection refers to the duration of hospitalization required for treatment of the CRE infection after diagnosis. Duration of ICU stay refers to the duration of ICU stay required for treatment of the CRE infection

## Discussion

CRE pathogens are reported continuously in recent years, but the type of infection and pathogen species were not the same, thus effective treatment has not yet formed [14, 15]. Deaths caused by an increase in CRE infection have been reported in a variety of susceptible populations such as children, cancer and burns [3, 16, 17]. In the Maryland National Institutes of Health report, carbapenem-resistant *K. pneumoniae* emerged in the clinical setting caused an outbreak in 18 patients, 11 of which died [18]. A report of hospital in Beijing showed a high mortality rate, 98 cases of CRE infection were studied. Pathogenic strains including KPC, IMP, NDM and other genotypes have a high mortality rate (57.4%) after infection [14]. In contrast, the data showed a lower mortality rate (< 30%) in our study than previously reported data. The main reason is due to lower resistance to quinolone antibacterial drugs, which are effective in treating many infected patients. The sensitivity to quinolones has not been reported in other reports, and the specific resistance may be related to the production of IMP metalloenzymes. But other cases of CRE infection with IMP enzyme did not appear the same drug [19–21], indicating that resistance of CRE to carbapenem involve many complex multi-drug resistance factors other than carbapenemase. In addition, some patients who were treated with carbapenems were still cured due to lack of an ideal antimicrobial drug. This effect may be explained on the basis of PK/PD principles and clinical adjuvant interventions. Proper nutritional support and timely removal of sources of bacterial infection such as drainage or ventilator are beneficial for the recovery of patients.

Several studies have investigated the clinical characteristics and outcomes of CRE-infected patients, who were usually elderly, physically weaker, and had multiple complications including diabetes and immunosuppressive [3, 17]. Our analysis confirmed the same situation, in which elderly patients are a significant contributor to CRE infection and newborns in the neonatal ICU and other pediatric wards are also susceptible to infection. Such patients were often exposed to the hospital environment and invasive operations, and suffering from serious illness, need special care. Our analysis confirmed that CRE had spread in different types of sites, such as the respiratory tract, urinary tract, bacteremia and surgical wounds. It has been reported infection CRE were highly correlated with indwelling catheter and mechanical ventilation, which was an important cause of related infection [22–24]. The findings of our analysis confirmed urinary tract infections were more concentrated in patients with urological tumors, and infections associated with local tissue damage involving SWI, BTI, DWI and SBFI were more present in surgical department. So strict disinfection and isolation measures to block the transmission of infection between patients is particularly important.

Treatment problems were mainly found in the treatment of CRE infection with only a few antimicrobial options, including polymyxin, aminoglycosides, tigecycline and fosfomycin [25, 26]. In the possible combination regimen, the effect of carbapenem treatment remains to be determined, such as high-dose infusion or double carbapenem treatment [27, 28]. Now the practical problem is that health care workers will face a rapid increase number of CRE infected patients, which may require special infection control measures and strict compliance. Moreover, the development of new antimicrobial agents and the use of traditional antimicrobic should be a recommended and effective method.

## Conclusions

In summary, we identified a large number of cases of CRE infection in 7 years from different parts, including site of infection, type of infection pathogens, risk factors, etc. Most of these pathogens have been confirmed to produce IMP-type carbapenemases, and retrospective analysis of cases of such bacterial infections will help to control future infections of these pathogens. Despite the high mortality rate, we still found that the selection of quinolone antibiotics can be effective in the treatment of CRE producing IMP type enzymes. The report on the treatment of Enterobacteriaceae producing IMP-type metallo-beta-lactamase is not common, our report is conducive to infection antimicrobial selected to provide a basis for the empirical treatment.

## Abbreviations

CRE: carbapenem-resistant *Enterobacteriaceae*
VABP: ventilator-associated bacterial pneumonia
cUTI: complicated urinary tract infection
AP: acute pyelonephritis
HABP: hospital-acquired bacterial pneumonia
SWI: superficial wound infection
BTI: biliary tract infection
DWI: deep wound infection
SBFI: sterile body fluids infection
CLSI: Clinical and Laboratory Standards Institute
SBP: systolic blood pressure
DIC: disseminated intravascular coagulation
ICU: intensive care unit
PFGE: pulsed field gel electrophoresis
